# Optically Active Plasmonic Metasurfaces based on the Hybridization of In-Plane Coupling and Out-of-Plane Coupling

**DOI:** 10.1186/s11671-018-2564-8

**Published:** 2018-05-10

**Authors:** Dong Wu, Liu Yang, Chang Liu, Zenghui Xu, Yumin Liu, Zhongyuan Yu, Li Yu, Lei Chen, Rui Ma, Han Ye

**Affiliations:** 1 State Key Laboratory of Information Photonics and Optical Communications, Beijing University of Posts and Telecommunications, Beijing, 100876 China; 2 School of Science, Beijing University of Posts and Telecommunications, Beijing, 100876 China

**Keywords:** Plasmonics, Metamaterials, Optical switching devices, Optical sensing and sensors, Subwavelength structures, Nanostructures

## Abstract

Plasmonic metasurfaces have attracted much attention in recent years owing to many promising prospects of applications such as polarization switching, local electric field enhancement (FE), near-perfect absorption, sensing, slow-light devices, and nanoantennas. However, many problems in these applications, like only gigahertz switching speeds of electro-optical switches, low-quality factor (Q) of plasmonic resonances, and relatively low figure of merit (FOM) of sensing, severely limit the further development of plasmonic metasurface. Besides, working as nanoantennas, it is also challenging to realize both local electric FE exceeding 100 and near-perfect absorption above 99%. Here, using finite element method and finite difference time domain methods respectively, we firstly report a novel optically tunable plasmonic metasurface based on the hybridization of in-plane near-field coupling and out-of-plane near-field coupling, which provides a good solution to these serious and urgent problems. A physical phenomenon of electromagnetically induced transparency is obtained by the destructive interference between two plasmon modes. At the same time, ultrasharp perfect absorption peaks with ultra-high Q-factor (221.43) is achieved around 1550 nm, which can lead to an ultra-high FOM (214.29) in sensing application. Particularly, by using indium-doped CdO, this metasurface is also firstly demonstrated to be a femtosecond optical reflective polarizer in near-infrared region, possessing an ultra-high polarization extinction ratio. Meanwhile, operating as nanoantennas, this metasurface achieves simultaneously strong local electric FE(|*E*_loc_|/|*E*_0_| > 100) and a near-perfect absorption above 99.9% for the first time, which will benefit a wide range of applications including photocatalytic water splitting and surface-enhanced infrared absorption.

## Background

Plasmonic metasurfaces, as two-dimensional versions of metamaterials, have a wide range of promising phenomena and applications including polarization switching [1], beam rotator [2], Fano resonance [3–7], nanoantennas [8–10], negative refractive index [11, 12], near-perfect absorbers [13–15], and invisibility cloaking. Especially, much attention has been paid to studying of electromagnetically induced transparency (EIT) phenomenon and Fano resonance based on plasmonic metasurfaces owing to many potential applications such as surface-enhanced Raman scattering (SERS) [3], surface-enhanced infrared absorption (SEIRA) [16], refractive index sensing [17–21], and quantum information storages. The concepts of EIT and Fano resonance both are originally discovered in quantum system. EIT is obtained by the destructive interference between two plasmon modes in classic system. Then, if the EIT is generated when a narrower plasmon mode destructively interferes with a broader plasmon mode, the resulting spectrum will have a Fano line shape. Zhang et al. firstly realized Fano resonance and EIT in a plasmonic nanostructure with a bright and dark element in one plane [22]. However, for most of reported plasmonic metasurfaces based on in-plane plasmonic coupling operating in a visible or near infrared (NIR) region, the coupling strength is determined by the accurate size of gap between resonant elements, but achieving precise, sub-10-nm gaps still is a challenge due to the limitations of current fabrication technology [8]. But these nanostructures strongly rely on the tiny inter-particle distance, which is not favorable to the large-area production. Different from the metasurface based on in-plane coupling effect, Liu et al. experimentally demonstrated plasmonic EIT using vertical stacking of the metamaterial elements for the first time [23]. Subsequently, a number of metamaterials (or metasurfaces) based on the planar or vertical design of plasmonic nanostructures are recently proposed and demonstrated to achieve EIT-like phenomena and Fano resonances [24–35]. Amin et al. demonstrated the asymmetric Fano-like spectral line-shape and a narrow EIT window in the response of the resonator constructed using both the gold frame and the graphene patch in one plane [17]. However, the quality factor of the Fano resonance in this metal structure is very low owing to optical losses in metal that cause significant broadening of the plasmonic resonances, which also is an extremely common problem in plasmonic nanostructures using metals [36–42]. To our knowledge, the Q-factors of most reported Fano resonances at visible and NIR region are generally lower than 10 [36–43]. Recently, Dayal et al. demonstrated a whispering-gallery-mode-based metallic metasurfaces realizing high Q (reaching 79) plasmonic Fano resonances at NIR frequencies [5]. However, this reported Fano resonance can only be achieved at a specific wavelength, which also is another common problem seriously restricting the further developments and applications of the Fano resonance or EIT phenomena. The active manipulation of Fano resonance or EIT window is highly desirable for many practical applications [19, 21, 35, 43]. Xia et al. designed and numerically demonstrated a tunable PIT system composed of sinusoidally curved and planar graphene layers, which can avoid any of the patterns of the graphene sheet [44]. In 2017, Yang et al. experimentally achieved a highly controllable absorption resonance with high-quality factor, which are firstly demonstrated to be a femtosecond optical polarization switching based on a plasmonic metasurface in a mid-infrared region [1]. Besides, a maximum electric field enhancement reaching 41.8 is also observed in this work. It is desirable to employ plasmonic nanoantennas that result in not only “hot spots” with a large local field enhancement but also a near-perfect absorption. Although tremendous progress in the exploration of enhancing the local electric field enhancement and improving the absorption, achieving strong local electric field enhancements (|*E*_loc_|/|*E*_0_| > 100) and near-perfect absorption (> 99%) simultaneously still remains a challenge, which will benefit a wide range of applications including plasmonic sensors, photocatalytic water splitting, SERS, and SEIRA. On the other hand, except for the polarization switching reported by Yang et al. [1], most traditional polarization-selective devices, such as waveplates and polarizers based on electro-optical effects, are either static or operating with only gigahertz switching speeds, which are limited by the required electronics [45, 46]. Thus, for the phenomena or applications of EIT effect, Fano resonance, and plasmonic nanoantennas based on a plasmonic metasurface, most of previously reported works usually suffer from these serious and urgent problems: (i) the broadening of plasmonic resonances owing to large optical losses in metals [5]; (ii) unadjustable operating wavelength of EIT effect or Fano resonances [35]; (iii) the challenge of achieving strong local electric field enhancements (|*E*_loc_|/|*E*_0_| > 100) and near-perfect absorption (> 99%) simultaneously [8]; (iv) generally, only gigahertz switching speeds of polarization-selective devices operating in visible or NIR region [1].

In this work, using finite difference time domain (FDTD) and finite element method (FEM) respectively, we propose and numerically demonstrate an optically active plasmonic metasurface based on the hybridization of in-plane coupling and out-of-plane coupling. In this metasurface system, the EIT-like effect can be achieved by breaking the structure symmetry, and the operating wavelength of the EIT widows can be tuned by changing the refractive index of the CdO layer, which can be optically controlled by tuning the pump light [1]. In this EIT-like reflection spectrum, a high Q-factor plasmonic resonance is obtained at a wavelength of 1550 nm, which is much higher than that of previously reported works [36–43]. Particularly, owing to the polarization independence of the metasurface, this plasmonic metasurface using In-doped cadmium also can function as a femtosecond polarization switch for TM-polarized light at 1550 nm. By tuning the pump light, we spectrally redshift the plasmonic resonances, and the metasurface achieves a large modulation depth of the reflection of the TM-polarized light from 0.003 to 60%, while maintaining a near-one reflection for the TE-polarized wave. To our knowledge, such a large modulation depth is far higher than those of previously reported plasmonic switch systems [47–55]. Note that the femtosecond polarization switch is firstly numerically demonstrated based on the plasmonic metasurface via the hybridization of in-plane coupling and out-of-plane coupling. At the same time, this metasurface can achieve near-perfect absorption above 99.9% and maximum electric field enhancement reaching 108 simultaneously, and the strong electric enhancement is confined within a circular area with a diameter of only 3 nm, which is very beneficial to single molecule detection for many surface-enhanced spectroscopies. Besides, owing to the sensitivity of refractive index changing and the ultra-sharp plasmonic resonance, this metasurface also can work as an ultra-high figure of merit (FOM) refractive index sensor.

## Methods

**Fig. 1:**
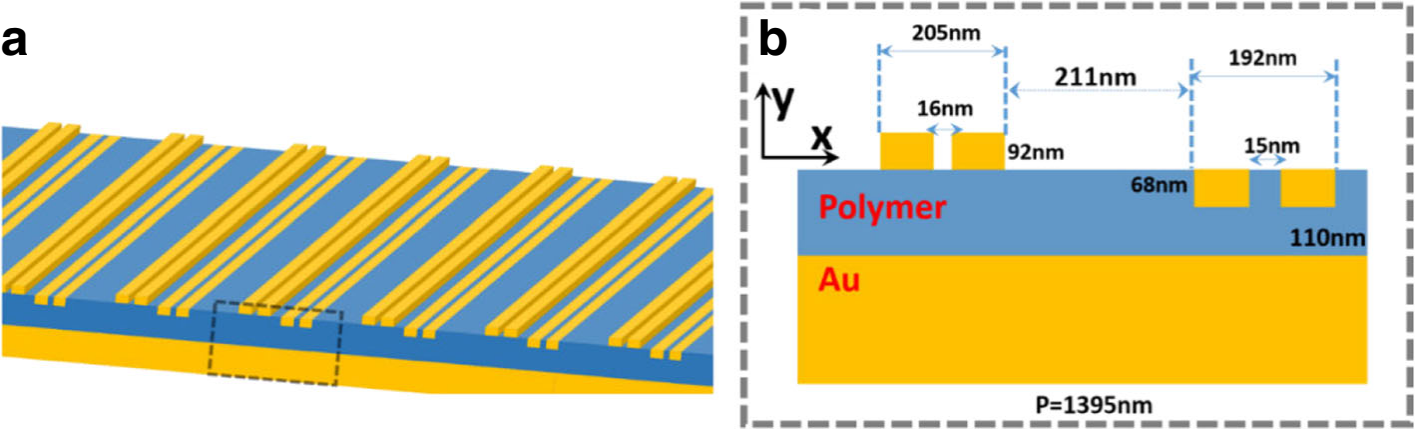
**a** Schematic structure of the proposed metasurface. **b** Cross section of the plasmonic metasurface with the geometric parameters

The proposed metasurface is schematically shown in Fig. 1a. Figure 1b presents the cross section of one unit cell of the metasurface with geometric parameters, which consists of two groups of gold bars and a polymer layer. Each group has two gold bars separated by a nanoslit. One group of gold bars is placed on the polymer layer, and the other group of gold bars was embedded in the polymer layer. The asymmetric gold nanobar array is periodically arranged on the thick gold substrate with a periodicity of *P* = 1395 nm. The proposed metasurface is illuminated by a normally incident transverse-magnetic (TM) light (the magnetic component perpendicular to the incident light). In this calculation, to ensure the reliability and accuracy of the simulated results, we employ FDTD and FEM methods to calculate the optical properties and electromagnetic field distributions of the proposed metasurface, respectively. The FEM calculation is performed by commercial software COMSOL MULTIPHYSICS. The period boundary condition is applied in the *x* direction and we set the perfectly matched layer (PML) on the boundary of the *y* direction. The mesh size is 0.8 nm in both *x* and *y* directions. The permittivity of Au is described by the Drude model, and the refractive index of the polymer is 1.5 [36, 56, 57]. The simulation background is assumed in air with *n*_air_ = 1. The absorption is given by *A* = 1 − *R*, owing to an opaque Au substrate (*T* = 0) [58].

## Results and Discussion

**Fig. 2:**
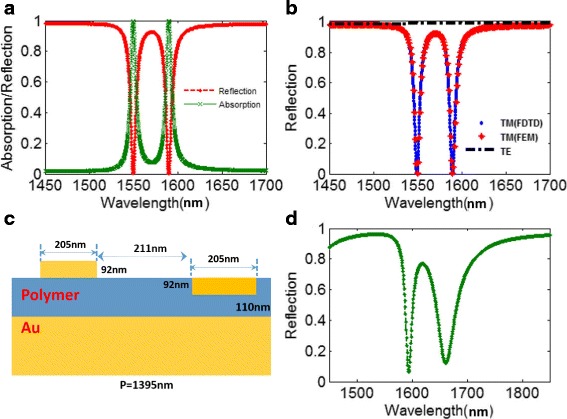
**a** Absorption and reflection spectra of the plasmonic metasurface shown in Fig. 1. **b** The reflection spectra of the metasurface shown in Fig. 1 calculated by FDTD and FEM, respectively. **c** The plasmonic metasurface composed of two gold bars with different distances from gold substrate. **d** Reflection spectrum of the plasmonic metasurface shown in **c**

As shown in Fig. 2a, we calculate and depict the reflection and absorption spectra of the proposed metasurface around 1550 nm at normal incidence under TM-polarized light. For the absorption spectrum, there are two distinct absorption peaks located at 1550 and 1588 nm with a near-perfect absorption efficiency above 99.9%, respectively. From the reflection spectrum shown in Fig. 2b, we observe an EIT-like spectral response of this metasurface in this wavelength range, and the same results of reflection spectra are demonstrated by using FDTD and FEM, respectively. The reflection spectrum of the proposed metasurface under TE polarization (the electric component perpendicular to the incident plane) is also presented in Fig. 2b with a black line, and the reflection is close to one indicating no absorption occurs in this metasurface for TE polarization. The polarization dependence of this metasurface can be easily explained by the asymmetric design of the proposed metasurface. Therefore, this metasurface couples efficiently for TM polarization and remains dark for TE polarization.

**Fig. 3:**
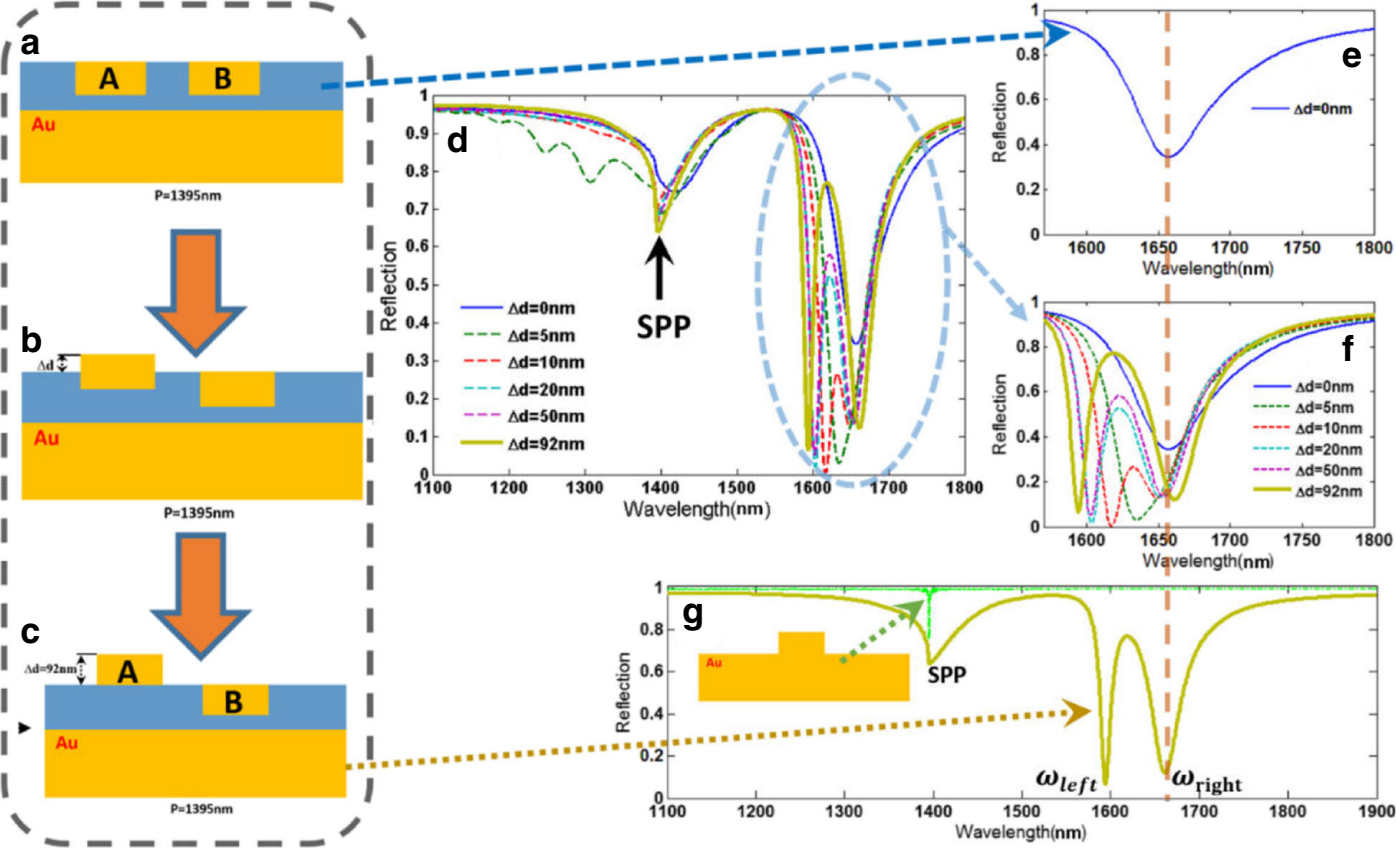
**a**–**c** The symmetry-breaking process of the plasmonic metasurface shown in Fig. 2c. **d** The reflection spectra of the plasmonic metasurface with various *∆d* at the wavelength range of 1100–1800 nm. **e** The reflection spectrum of the plasmonic metasurface with *∆d* = 0 at the wavelength range of 1550–1800 nm. **f** The reflection spectra of the plasmonic metasurface with various *∆d* at the wavelength range of 1550–1800 nm. **g** The reflection spectra of the plasmonic metasurface with various *∆d* = 92 nm and the all metal structure at the wavelength range of 1100–1800 nm, respectively

To easily explain the EIT-like phenomenon of the proposed metasurface in Fig. 2a, we initially consider a relatively simple metasurface without nanoslit shown in Fig. 2c, which is composed of two gold nanobars with different distances from gold substrate. The reflection spectrum of this metasurface without nanoslit is calculated and depicted in Fig. 2d. Clearly, an EIT-like spectral response with an asymmetric line shape emerges, which may be due to the coupling effect between the two gold bars. Then, the symmetry-breaking process (Fig. 3a-c) of the structure is investigated to clarify underlying forming process of the EIT-like window. The variations of the reflective spectra with changing *∆d* are calculated and depicted in Fig. 3d. For *∆d* = 0, there is only one reflection dip around 1653 nm in the working waveband, as shown in Fig. 3e. As *∆d* increases, we notice that there appear the EIT-like spectral response with two reflection dips (*ω*_Left_ and *ω*_Right_). If further increasing *∆d*, the *ω*_Left_ mode can be further enhanced, and these calculated results indicate that the *ω*_Left_ mode may be very relevant to the gold nanobar A. At the same time, with increasing *∆d*, the resonance wavelength of *ω*_Left_ mode show slight red shift, and the resonance wavelength of *ω*_Right_ mode remains almost no change around 1653 nm. Through the above analysis, the generation of EIT-like phenomena can be contributed to the asymmetry of the nanostructure. Besides, to interpret the plasmonic resonance at 1395 nm in the reflection spectra shown in Fig. 3d, g, the reflection spectra are compared between the designed metasurface and metallic grating structure (see insert of Fig. 3g). For the metallic grating structure, there is also a resonance dip at 1395 nm, resulting from the excitation of surface plasmon polariton (SPP) from previously reported studies [58, 59]. Thus, the plasmonic resonance of this metasurface at 1395 nm is caused by the excitation of SPP.

**Fig. 4:**
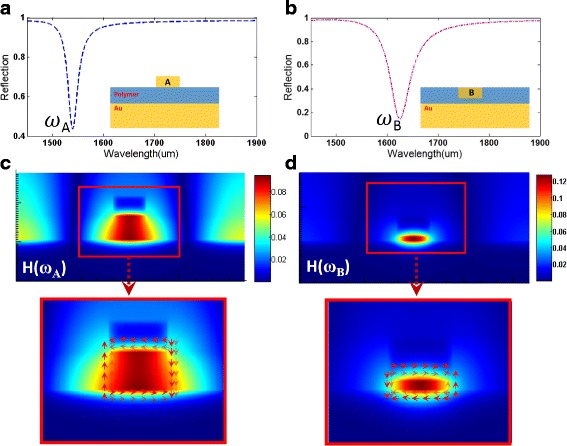
**a** Reflection spectrum of the plasmonic metasurface with only the gold nanobar A. **b** Reflection spectrum of the plasmonic metasurface with only the gold nanobar B. **c** Calculated magnetic field distributions H of the metasurface at resonant wavelengths of *ω*_A_ mode. **d** Calculated magnetic field distributions H of the metasurface at resonant wavelengths of *ω*_B_ mode. (The thickness of both gold A and gold B is 92 nm; the width of both gold A and gold B is 92 nm; the thickness of polymer is 110 nm; the period is 1395 nm)

**Fig. 5:**
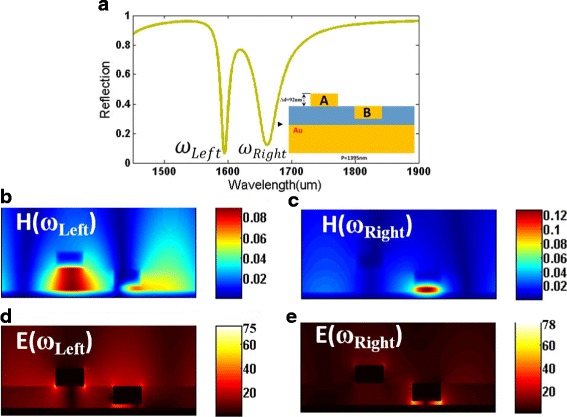
**a** Reflection spectrum of the plasmonic metasurface shown in Fig. 2. **b**, **c** Calculated magnetic field distributions H of the metasurface at resonant wavelengths of the *ω*_Left_ mode and *ω*_Right_ mode, respectively. **d**, **e** Calculated electric field distributions (|*E*_loc_|/|*E*_0_|) of the metasurface at resonant wavelengths of the *ω*_Left_ mode and*ω*_Right_ mode, respectively

Then, we also respectively investigate the reflection spectra of the metasurface constructed using film-coupled nanobar systems with only the gold nanobar A and the gold nanobar B, as shown in Fig. 4a, b. When excited with TM incident light separately, a narrower plasmon mode (*ω*_A_) is excited in the metasurface with gold nanobar A, and a broader plasmon mode (*ω*_B_) is observed in the metasurface with gold nanobar B. To more clearly illustrate the physical mechanism behind these two plasmon modes, we respectively calculate the magnetic field distributions at these two reflection dips, as shown in Fig. 4c, d. The red arrows present currents whereas the color map presents the magnitude of the magnetic field. For the *ω*_A_ mode shown in Fig. 4a, it can be observed that the magnetic field is confined to the gap between the gold nanobar A and the gold substrate. Besides, the antiparallel currents are observed at top and bottom internal metallic interfaces. Therefore, the plasmon mode is primarily associated with magnetic resonance caused by circulating currents, and the incident light energy is dissipated by the ohmic loss of metal, causing the reflection dip in *ω*_A_ mode. Then, for the *ω*_B_ mode in Fig. 4b, the circulating currents are in the opposite direction to that of the currents of the *ω*_A_ mode, which also can excite the magnetic resonance. For the film-coupled nanobar system with both gold nanobar A and the gold nanobar B, the phenomenon in Fig. 5a also can be treated as double Fano resonances with two reflection dips (*ω*_Left_ and *ω*_Right_) owing to the asymmetric line shape [3]. This asymmetric Fano-like spectral line shape and an EIT-like window is obtained from the destructive interference between the narrower plasmon mode (*ω*_A_) shown in Fig. 4a and the broader plasmon mode (*ω*_B_) shown in Fig. 4b. To our knowledge, Fano resonances are first observed in artificially structured arrays of same shaped resonators with asymmetrical positions.

To further explore the physical mechanism behind these two plasmonic resonances (*ω*_Left_ and *ω*_Right_) shown in Fig. 4c, the magnetic field H and electric field |*E*_loc_|/|*E*_0_| distributions at the wavelengths of these two resonances are calculated and depicted in Fig. 5. On the one hand, according to Fig. 5b, c, the magnetic fields are mainly localized at the dielectric layer between the gold nanobars and the gold substrate, which is the key feature of the out-of-plane coupling between the gold bars and the Au substrate. Clearly, different field distributions are observed for these two resonances excited at two absorption peaks. For the *ω*_Left_ mode, the magnetic field is localized at the gap between the gold nanobar A and the gold substrate, indicating that the *ω*_Left_ mode is closely related to the out-of-plane coupling between the gold nanobar A and the gold substrate, which is similar but not the same as the magnetic field of the *ω*_A_ mode in Fig. 4c owing to the coupling between the *ω*_A_ mode and *ω*_B_ mode. For the*ω*_Right_ mode, the magnetic field is localized at the nanogap between the gold nanobar B and the substrate. Therefore, the *ω*_Right_ mode is mainly contributed to the out-of-plane coupling between the gold nanobar B and the gold substrate. On the other hand, the electric fields are strongly enhanced and localized in an ultrasmall area at the edges of the gold bars. Then, except for the physical phenomena of EIT, this metasurface also can be treated as plasmonic nanoantennas (PNs), confining the free-space incident lights into sub-wavelength region with the local field enhancement, which is a very important and fundamental research for nanophotonic systems. Here, the factor |*E*_loc_|/|*E*_0_| is defined to evaluate the performance of local electric field enhancements of PNs. As shown in Fig. 5d, e, the local electric field enhancements of the metasurface can reach as high as 75. However, although local electric field enhancements are achieved using film-coupled nanobar systems, according to Fig. 4c, there is still a considerable amount of work to be done to realize a near-perfect absorption, which results in a small modulation depth. From previous researches [8], we know that achieving both large local electric field enhancement and near-perfect absorption will benefit a wide range of applications, including plasmonic sensors, photocatalytic water splitting, SERS, and SEIRA. Besides, this metasurface structure shows a relatively broader linewidth. Because the Q-factor of plasmonic resonance is defined as Q = λ/full width at half maximum (FWHM), a broader resonance will lead to a lower Q plasmonic resonance. Therefore, the broad FWHM and small modulation depth of those resonances may hamper applications such as refractive index sensing, polarization switching, and slowing down light, where a sharp spectral response is desired.

**Fig. 6:**
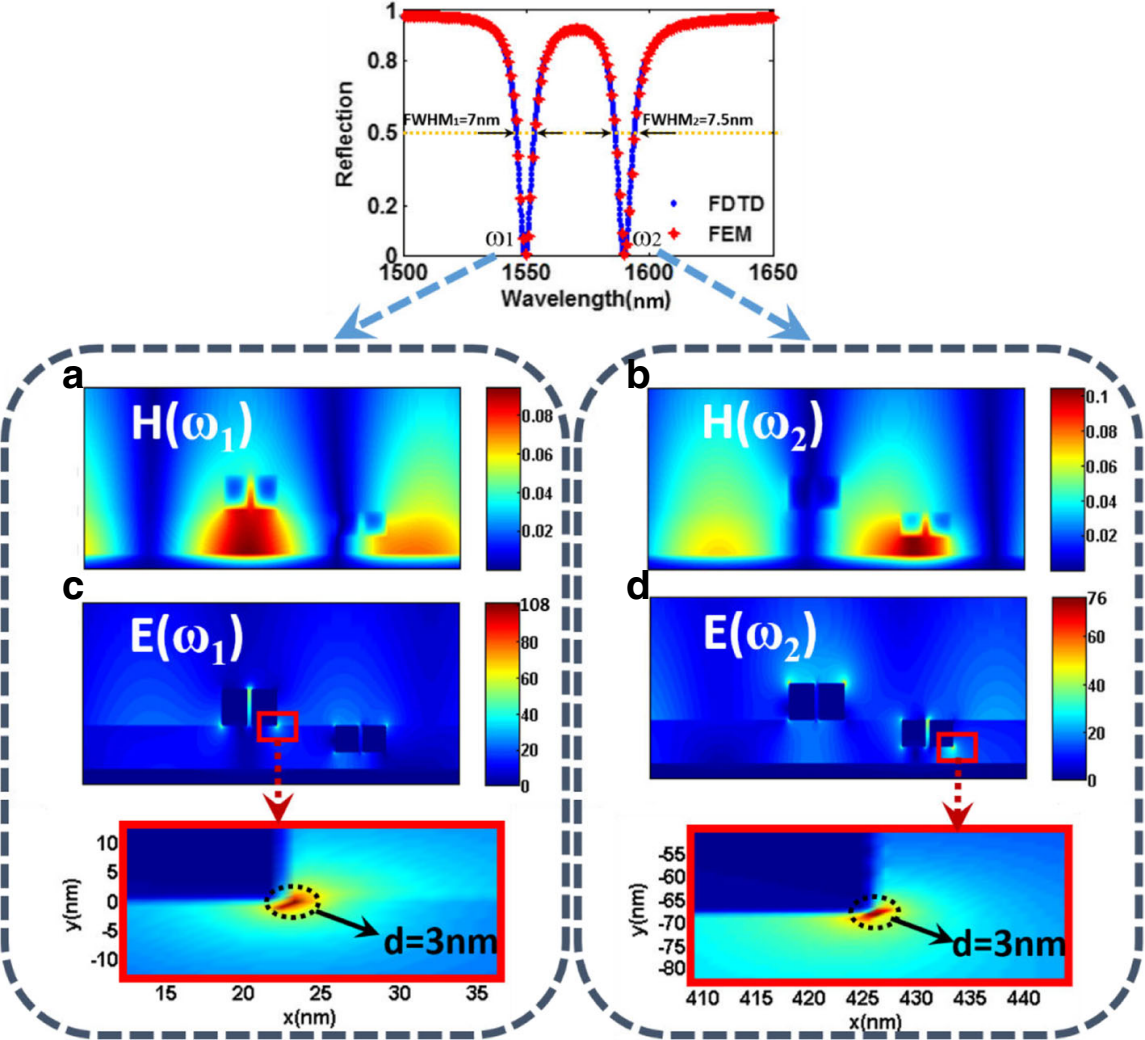
**a**, **b** Calculated magnetic field distributions H of the metasurface at resonant wavelengths of 1550 and 1588 nm, respectively. **c**, **d** Calculated electric field distributions (|E_loc_|/|*E*_0_|) of the metasurface at resonant wavelengths of 1550 and 1588 nm, respectively

To simultaneously realize large local electric field enhancement, near-perfect absorption, and high Q-factor resonance, here we introduce the concept of the hybridization of out-of-plane plasmon coupling and in-plane plasmon coupling in this work. Clearly, compared with the film coupled nanobar metasurface based on out-of-plane coupling, this proposed metasurface in Fig. 1 has superior absorption properties as shown in Fig. 2. Particularly, the FWHM of the plasmonic resonance at 1550 nm is 7 nm, resulting in a Q-factor (Q = *λ*/FWHM = 1550 nm/7 nm) of 221.43, which is much higher than those of previously reported works [36–42]. Then, in order to gain further physical insights into the high-Q Fano resonances and the perfect absorption arising from the original metasurface in Fig. 1, we plot the simulated magnetic and electric field distribution at resonance wavelengths of 1550 nm (*ω*_1_) and 1588 nm (*ω*_2_), as shown in Fig. 6. Clearly, the magnetic field is mainly located in the gap between the gold bar and the gold substrate, and part of magnetic field is propagated to the nanosilt between two gold nanobars. Different from the electric field only resulting from the out-of-plane coupling as shown in Fig. 5d, e, the electric field of this proposed metasurface is also strongly localized within an ultrasmall area between the two gold bars according to Fig. 6c, d, which signifies the strong localized surface plasmon (LSP) coupling between the two gold nanobars. Figure 6c shows that the maximum electric field enhancement at the resonant wavelength can reach as high as 108, around 1.4 times compared to the only film-coupled metasurface shown in Fig. 5d, which is much higher than those of the previously reported nanoantennas [21, 60–65]. Particularly, we can clearly observe that the ultrasmall “hot spot” featured by the strong electric enhancement is confined within a circular area with a diameter of only 3 nm. Thus, these hybridized metasurface systems have been shown to simultaneously have superior absorption, large local electric enhancement, and small lateral resolution, which are very helpful for probing the accurate properties of single molecules for many surface-enhanced spectroscopies, due to their ability of supporting both the LSP and out-of-plane couplings.

**Fig. 7:**
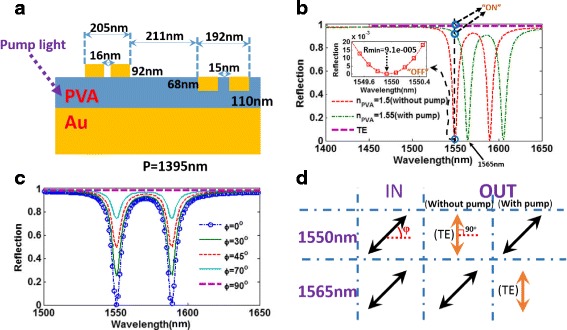
**a** Schematic structure of the proposed metasurface with pump light. **b** The reflection spectra of the proposed metasurface with TM- and TE-polarized incident light, with and without pump light. **c** The reflection spectra of the proposed metasurface with various polarization angles. **d** The projected output polarizations of the metasurface, with and without a pump, at 1550 and 1588 nm

From the analysis results in Fig. 2b, we know that the metasurface couples efficiently for TM polarization and remains dark for TE polarization, owing to asymmetric design, which has a potential application in polarization switch. Then, considering that the refractive index of PVA (poly(vinyl alcohol) can be changed with alteration of pump power [36, 56, 57], the operating wavelength of plasmonic resonances generally can be changed by varying the refractive index of dielectric layer. Then, Fig. 7a,b illustrates that the proposed metasurface can indeed work as a polarization switch, which is based on a reflective polarizer containing a tunable resonance for TM-polarized light by changing the refractive index of PVA. Clearly, as shown in Fig. 7b, without an external stimulus, the TM-polarized light is completely absorbed at wavelength of 1550 nm (“off” state), and this metasurface can completely reflect the TM-polarized light at wavelength of 1565 nm (“on” state). With an external stimulus, the Fano resonance for the TM-polarized wave is shifted to 1565 nm (“off” state), and this metasurface becomes completely reflective for TM-polarized light at 1550 nm (“on” state). Clearly, in Fig. 7b, this metasurface can realize a reflection value change from 0.009 to 98% at 1550 nm, and such a large modulation depth is far higher than previously reported plasmonic switch systems. On the other hand, according to Fig. 7b, the reflection of the incident light remains close to one with and without the external stimulus for TE polarization (“on” state). Therefore, this metasurface can realize a polarization switch for TM-polarized light based on a reflective polarizer with an extinction ratio of 11,000 (*R*_TE_/*R*_TM_ = 0.99/0.00009 = 11,000) at 1550 nm. We also give a calculation about the effect of polarization angle *φ* on the reflection spectra, as shown in Fig. 7c. Clearly, the absorption performance will gradually deteriorate at the resonant wavelengths with increasing *φ*, which can be explained by that the incident electric field E can be decomposed into TE- and TM-polarized light and the TE-polarized light is reflected. Based on the calculated results in Fig. 7c, the projected output polarizations of the metasurface, with and without a pump, at 1550 and 1588 nm are plotted in Fig. 7d.

**Fig. 8:**
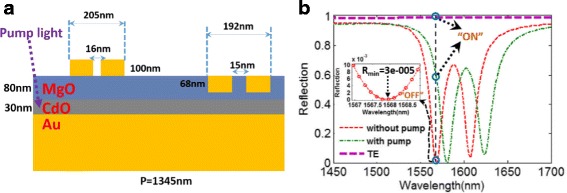
**a** Schematic structure of the CdO-based metasurface with pump light. **b** The reflection spectra of the CdO-based metasurface with TM- and TE-polarized incident light, with and without pump light

In-doped CdO is one kind of optically tunable plasmonic material, and the femtosecond polarization switch operating at 2.8 μm based on the photoexcited CdO film has been demonstrated experimentally in the recent literature [1]. In order to further improve the tunable capacity of our EIT structure, we investigate the optical properties of the proposed metasurface using CdO [1]. The schematic structure of the CdO-based metasurface with geometric parameters is depicted in Fig. 8a. The refractive index of MgO and CdO is obtained from references [1, 66], respectively. According to Fig. 8b, we show the reflection spectra with and without a pump around 1568 nm. In the static “on” state, the proposed metasurface is a polarizer that reflects the TE-polarized wave and completely absorbs the TM-polarized wave at wavelength 1568 nm. In the static “off” state, the proposed metasurface becomes reflective for both TM and TE polarizations at 1568 nm, and the resonance for TM-polarized wave is shifted to 1581 nm, owing to the refractive index change of In-doped CdO by an external stimulus. Particularly, this reflective polarizer can achieve a huge extinction ratio at 1568 nm for TM-polarized light owing to the extremely low *R*_min_ shown in Fig. 8b. The huge extinction ratio of the CdO-based metasurface make it a good platform for active polarization control. Note that, the refractive index of the CdO can be tuned by changing pump power, which also can realize active control of the operating wavelength of the EIT-like effect. Besides, we can find that the pump light has no influence to the other materials (including gold, MgO), which have been demonstrated by experiments in these references [1, 36, 56, 57].

**Fig. 9:**
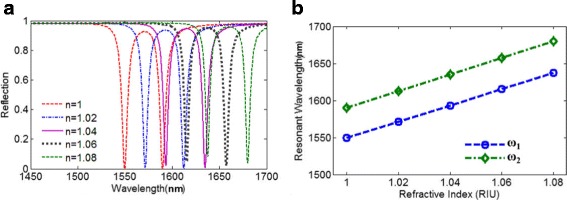
**a** Reflection spectra of the proposed metasurface with varying refractive index of surrounding environment. **b** Resonant wavelengths of the proposed metasurface as a function of the surrounding refractive index

Besides, for the sensitivity of refractive index from the above analysis, the proposed metasurface also can be applied to detect the change of refractive index of surrounding environment. In many previously reported works about refractive index sensing, the light intensity of reflection/transmission wave is usually measured when the surrounding refractive index is variable with a specific operating wavelength. Then, to demonstrate the sensing property of this metasurface, Fig. 9 presents that the double plasmonic resonances are red-shifted with the increasing of surrounding refractive index changes. With the variation of the surrounding refractive index, the sensitivity(S) can reach S = 1500 nm/RIU. Then, the FWHM of the reflection dip at ω_1_ and ω_2_ is 7 and 7.5 nm respectively, which indicate that this metasurface can operate as an ultra-high FOM(S/FWHM_1_ = 214.29) refractive index sensor in the near infrared region. The FOM = 214.29 is much higher than those of most previously reported plasmonic refractive index sensor [58, 67–70].

## Conclusions

In this work, a novel optically tunable hybridized metasurface is proposed and exploited to generate the EIT-like phenomena around 1550 nm, which hybridizes the in-plane near-field coupling between gold nanobars and the out-of-plane near-field between gold nanobars and substrate. For the traditional design of EIT-like metamaterials, two different shaped resonators, in planar or vertical arrangement, are working as bright mode and dark mode respectively, which can induce EIT effect by bright-dark mode coupling. However, in this structure, the two individual bright modes mainly result from the two same shaped resonators with different positions, which is neither a planar structure nor a vertical structure. The resulting two fundamental plasmon modes of the hybridized system are also investigated in detail. By introducing indium-doped CdO, the operating wavelength of the EIT-like phenomenon can be tuned optically. At the same time, this metasurface is firstly demonstrated to be a femtosecond polarization switch for TM-polarized light at 1550 nm, which can realize an extinction ratio (*R*_TE_/*R*_TM_) much higher than that of previously reported polarization switches. Besides, operating as plasmonic nanoantennas, this metasurface also achieves a strong local field enhancement (|*E*_loc_|/|*E*_0_| > 100) and a near-perfect absorption (> 99%) simultaneously. Owing to these above advantages, this proposed metasurface is a promising candidate for femtosecond polarization switching, plasmonic nanoantennas, and high FOM refractive index sensor.

## Abbreviations

EIT: Electromagnetically induced transparency
FDTD: Finite difference time domain
FE: Field enhancement
FEM: Finite element method
FOM: Figure of merit
FWHM: Full width at half maximum
PML: Perfectly matched layer
SEIRA: Surface-enhanced infrared absorption
SERS: Surface-enhanced Raman scattering
